# Video capsule endoscopy as the initial examination for overt obscure gastrointestinal bleeding can efficiently identify patients who require double-balloon enteroscopy

**DOI:** 10.1186/s12876-015-0362-7

**Published:** 2015-10-14

**Authors:** Yoshimasa Maeda, Kosaku Moribata, Hisanobu Deguchi, Izumi Inoue, Takao Maekita, Mikitaka Iguchi, Hideyuki Tamai, Jun Kato, Masao Ichinose

**Affiliations:** Second Department of Internal Medicine, Wakayama Medical University, 811-1 Kimiidera, Wakayama City, Wakayama 641-0012 Japan

**Keywords:** Video capsule endoscopy, Overt OGIB, Double-balloon enteroscopy, Choice of route

## Abstract

**Background:**

Both double-balloon enteroscopy (DBE) and video capsule endoscopy (VCE) have similar diagnostic yields for patients with overt obscure gastrointestinal bleeding (OGIB). However, the choice of initial modality is still controversial. The aim of this study was to show the clinical outcome of the strategy of initial VCE, followed by DBE.

**Methods:**

Eighty-nine consecutive overt OGIB patients who had undergone VCE as the initial examination were analyzed. The interpreters of VCE evaluated the necessity of performing DBE, and the antegrade or retrograde route was chosen, depending on the transit time of the capsule.

**Results:**

Thirty-seven patients (42 %) underwent DBE depending on the findings of VCE. Of these, bleeding sites in the small bowel were identified in 29 patients with the initially selected route (21 antegrade and 8 retrograde). The remaining 8 later underwent DBE by the other route, but 7 had no bleeding lesion, which was confirmed by second-look VCE. One remaining patient had a jejunal varix found by VCE, but DBE from either side could not reach the lesion. The sensitivity and negative predictive value of VCE were 100 %, both for the presence of small bowel lesions and the requirement of hemostasis in the small bowel; this indicated that VCE never misses relevant findings in the small bowel, and that negative VCE findings correspond to the lack of necessity for further examination.

**Conclusions:**

VCE as the initial examination can efficiently identify overt OGIB patients who require DBE. The strategy of initial VCE for overt OGIB appears to be reasonable.

## Background

Obscure gastrointestinal bleeding (OGIB) is defined by the American Gastroenterological Association as bleeding of unknown cause after upper or lower endoscopy [1]. OGIB can be further classified into occult and overt bleeding. Occult OGIB is defined as iron deficiency anemia, with or without a positive fecal occult blood test, while overt OGIB indicates clinically perceptible bleeding that recurs or persists despite negative initial endoscopic and radiologic evaluations. In overt OGIB, therefore, immediate identification of the bleeding lesion is needed for appropriate therapy, including hemostatic treatment to rescue unstable patients. However, the diagnostic performance and accuracy of conventional diagnostic strategies, including small intestinal radiography, abdominal computed tomography, magnetic resonance imaging, digital subtraction angiography, radionuclide scanning, and intraoperative endoscopy, have not been satisfactory for detecting small intestine diseases [2, 3].

Since the introduction of video capsule endoscopy (VCE) [4] and double-balloon enteroscopy (DBE) [5] in 2000 and 2001, respectively, these modalities have enabled direct observation of the lumen of the small intestine. VCE is a new type of endoscopy, which allows painless endoscopic imaging of the entire small bowel, and is well tolerated by patients [6]. However, lack of therapeutic capability, including hemostasis, is a critical drawback of the device. On the other hand, DBE is a new insertion method for enteroscopy, which enables endoscopic scrutiny of the entire small intestine, and endoscopic intervention, such as polypectomy, hemostasis, and sampling of tissue. Total enteroscopy may be achieved through a combination of antegrade and retrograde approaches [7]. However, it is an invasive procedure, and it typically requires patient sedation, two endoscopists, and additional medical staff.

Both VCE and DBE reportedly demonstrate similarly high diagnostic yields of small intestinal disorders [8, 9]. However, in the clinical situation of overt OGIB, the role of these new modalities has not been fully established. In particular, the choice of initial modality is still controversial. The policy of some endoscopists is that DBE by the antegrade route should be the initial procedure for overt OGIB, because the intestinal lesions causing overt OGIB are most likely localized in the proximal part of the small bowel [10]. Others advocate that VCE should be the initial choice because it is noninvasive and is capable of visualizing the entire small intestine. Recently published guidelines in Europe recommended VCE as the initial examination for overt OGIB, mainly due to its safety [11]. However, the validation of this strategy is incomplete.

Our recent policy for overt OGIB is also initial VCE, followed by DBE via the antegrade or retrograde route, as appropriate. Hence, consecutive patients with overt OGIB underwent VCE as the initial examination for small bowel lesions in our hospital. In this study, the clinical outcome of these patients is shown, and the strategy of initial VCE for overt OGIB is validated.

## Methods

### Patients

This is a retrospective analysis of overt OGIB patients who had visited Wakayama Medical University Hospital between December 2008 and December 2014. During this period, 89 consecutive patients had undergone VCE as the initial examination for the bleeding site in the small intestine. All patients had ongoing or recent overt gastrointestinal (GI) bleeding, and had undergone upper endoscopy and colonoscopy with negative results. The ongoing bleeding was defined as visible melena at the time of first consultation to our institution. The remaining subjects were regarded as having previous OGIB. Some patients underwent upper endoscopy and/or colonoscopy at another institution.

The retrospective analysis was approved by the institutional review board of Wakayama Medical University. Written informed consent was obtained from each patient.

### VCE procedure

Patients were examined using PilCam SB® (SB1, SB2, or SB3) (Covidien, Irvine, CA, USA). The video capsule was swallowed after preparation with 50 g of magnesium citrate. The digital information recorded was downloaded into a computer, and the images were analyzed using the proprietary RAPID software (Covidien) by two experienced reviewers independently. When the diagnosis differed between the two reviewers, they conferred until they reached a consensus. The transit time through the entire small intestine was recorded. In addition, if the bleeding site was detected, the time when the capsule reached the site was also recorded.

### DBE procedure

The indication for DBE was determined by the VCE reviewers depending on the findings. If a suspected cause of bleeding, such as a polypoid lesion, open ulcer, vascular lesion, Dieulafoy’s lesion, or hemorrhagic erosion was detected by VCE, DBE was indicated. DBE was performed using an enteroscope for small bowel observation (EN-450 T5/W or EN-580 T, Fujinon Inc., Saitama, Japan), using the FUJIFILM high-resolution enteroscopy system.

The antegrade or retrograde insertion route was chosen according to the transit time of the VCE. If the suspected bleeding site was detected at a time point before one-half of the transit time through the small intestine, DBE was inserted orally. Alternatively, the device was inserted by the retrograde route. DBE was inserted with no further bowel preparation after VCE.

### Statistical analysis

Statistical analysis was conducted using the STATA program (version 13, Stata Corp., TX, USA). Sensitivity, specificity, positive predictive value (PPV), negative predictive value (NPV), and accuracy were determined with 95 % confidence intervals (CIs).

## Results

### Patient course and VCE results

During the study period, 89 patients with overt OGIB underwent VCE as the initial examination for the small intestine, and a record of the entire small intestine was obtained in all of the patients. The clinical data for these patients are shown in Table 1. Fifty-five (62 %) patients had ongoing bleeding, while the remaining 34 (38 %) had a recent history of overt bleeding. Medications associated with bleeding, including antiplatelet agents, anticoagulants, and/or nonsteroidal anti-inflammatory drugs, were used by 31 (35 %) patients. The drugs had been taken just before starting VCE in all patients. Because DBE was done within almost 24 hours after VCE, those drugs seemed to be active even during DBE.

**Table 1:** Clinical characteristics of study patients

Characteristic	Patients *n* = 89
Age, years, median (range)	70 (30–92)
Male : Female	48 : 41
Type of gastrointestinal bleeding	
Overt ongoing	55 (62 %)
Overt previous	34 (38 %)
Medication used	
Antiplatelet medicine	16 (18 %)
Anticoagulation	9 (10 %)
Both anticoagulation and antiplatelet	3 (3 %)
NSAIDs	3 (3 %)
Hemoglobin, g/L, median (range)	9.3 (5.3–14.7)

*NSAIDs*, Non-steroidal anti-inflammatory drugs

**Fig. 1:**
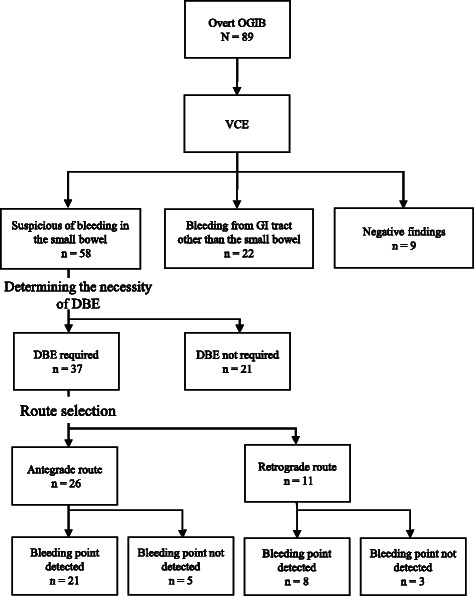
Flow chart of the study subjects

The clinical course of these patients is shown in Fig. 1. Bleeding in the small intestine could be observed in 58 (55 %) subjects with VCE. Bleeding from the GI tract at a site other than the small intestine, including gastric antral vascular ectasia, duodenal ulcer, and diverticulum of the colon, was detected in 22 (25 %). These 22 patients were later reexamined with upper endoscopy or colonoscopy, and the other bleeding site was confirmed. The remaining 9 patients had negative findings in the small bowel as well as in other GI segments with VCE, and the bleeding sites of these patients could not be elucidated during follow-up.

Of 58 patients with positive findings in the small bowel, 21 were not considered for DBE, because they had tiny erosive lesions (13), a submucosal-type lipoma (4), a single ulcer in a healing stage (2), radiation enteritis (1), or intestinal Behçet’s disease (1). Of these, 20 patients, except one with intestinal Behçet’s disease, required no further examination or intervention during follow-up. The Behçet’s disease patient had episodes of melena three times prior to examination by VCE, which revealed several punched-out ulcers in the small intestine. However, intestinal perforation requiring emergency enterectomy occurred in this patient, just after the VCE procedure, and before determination of the indication for DBE. Although the causal relationship between perforation in this patient and VCE procedure was uncertain, the event may be considered as the complication of VCE. Accordingly, the remaining 37 patients underwent DBE.

### Choice of route and the findings of DBE

**Fig. 2:**
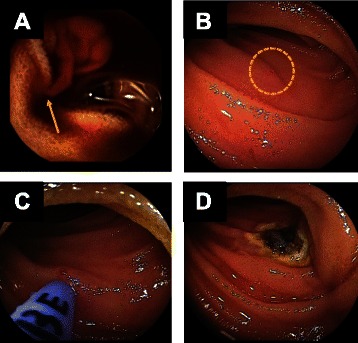
A case of overt ongoing OGIB. **a** VCE showed spurting bleeding from Dieulafoy lesion (arrow). **b** DBE was inserted perorally according to the transit time of VCE, and the bleeding point could be detected easily (circle). **c**, **d** Hemostasis was performed with argon plasma coagulation

Antegrade or retrograde choice of route for DBE was determined according to the recorded time to the bleeding site, as shown in the Methods section, resulting in 26 using the antegrade route and 11 the retrograde route. The bleeding lesions were found in 21 of 26 patients with the antegrade route, and hemostatic treatment was performed in 13. On the other hand, bleeding lesions were detected in 8 of 11 patients who had undergone DBE with the retrograde route, and hemostatic treatment was performed in 5. Figure 2 shows representative images of VCE and DBE of a patient with Dieulafoy lesion.

Bleeding lesions could not be identified by initial DBE with the antegrade route in 5 patients and in 3 patients with the retrograde route. All patients subsequently underwent DBE with the alternate route. However, the bleeding sites could not be found in any of these patients. One patient with type C liver cirrhosis was diagnosed with a jejunal varix with VCE. Antegrade DBE was initially carried out, but could not reach the varix because of adhesions after splenectomy. A retrograde approach for this patient then also failed to reach the lesion. For the remaining 7 patients, VCE was performed again three months later, but none showed positive findings in the small intestine.

### Diagnostic performance of VCE

The sensitivity, specificity, positive predictive value (PPV), negative predictive value (NPV), and accuracy of VCE, for the detection of both the bleeding site in the small intestine and the lesion requiring hemostasis, with the findings on DBE defined as the gold standard, are shown in Table 2. The performance of VCE was particularly good, with 100 % sensitivity and NPV in detection of both the bleeding site and lesions requiring hemostasis.

**Table 2:** Diagnostic performance of VCE for small intestine lesions and for lesions requiring hemostasis

	For small intestine disease	For lesions needed hemostasis
Sensitivity (%) (95 % CI)	100 (94.5–100)	100 (87.2–100)
Specificity (%) (95 % CI)	85.4 (78.9–85.4)	77.6 (73.4–77.6)
PPV (%) (95 % CI)	88.9 (84.0–88.9)	59.5 (51.8–59.5)
NPV (%) (95 % CI)	100 (92.4–100)	100 (94.6–100)
Accuracy (%) (95 % CI)	93.3 (87.3–93.3)	83.1 (76.8–83.1)

*PPV* positive predictive value, *NPV* negative predictive value, *CI* confidence interval

The comparison of the diagnoses between VCE and DBE in 37 patients is shown in Table 3. The diagnosis of 3 AVMs and 2 GISTs were confirmed by surgical specimens. Polyps revealed to be 2 lipomas and 1 hemangioma with reference to the resected specimens with endoscopic mucosal resection. VCE misdiagnosed 8 patients, based on a diagnosis with DBE. The diagnoses with DBE in 3 patients who had been regarded as having a Dieulafoy’s lesion with VCE, were diverticulum of the colon, diverticulum of the jejunum, and ulcer scarring. No significant findings were observed with DBE in 4 patients diagnosed as an erosion and 1 diagnosed as an ulcer.

**Table 3:** Consistency of the findings between VCE and DBE

	VCE findings	Diagnosis with DBE
Dieulafoy lesion	9	6^a^
Angiectasia	8	8
Erosion	6	2^b^
Ulcer	4	3^c^
Polyp	3	3
AVM	3	3
GIST	2	2
Jejunum varix	2	1^d^

AVM, Arteriovenous malformation ; GIST, Gastrointestinal stromal tumor

a, The diagnoses of 3 patients were diverticulum of the jejunum, diverticulum of the colon, and ulcer scarring

b, No significant findings were observed with DBE in 4 patients

c, No significant findings were observed with DBE in one patient

d, Jejunal varix was suspected in one patient, but DBE failed because of adhesions

## Discussion

Recent meta-analysis of examinations for small bowel lesions reported that both VCE and DBE have similarly high diagnostic yields [9]. However, the choice of modality for use in overt OGIB has not yet been clearly established. In particular, the choice of the initial approach is controversial. Recommendation of initial VCE by the European Society of Gastrointestinal Endoscopy is based only on the excellent safety profile of VCE, [11] and the validation of the superiority of the strategy is incomplete.

On this background, we demonstrated the results of our clinical experience for overt OGIB. Our policy for overt OGIB was to perform VCE initially, followed by DBE as necessary. Our results have the following clinically relevant implications. First, an initial VCE procedure could precisely determine the necessity of subsequent DBE, except one case with perforation with obscure causal relationship with VCE. In fact, the sensitivity and NPV of VCE were 100 %, both for the presence of small bowel lesions and the requirement of hemostasis in the small bowel. These perfect results indicate that VCE never misses relevant findings in the small bowel, and that negative VCE findings correspond to the lack of necessity for further examination. Because missed overt OGIB may lead to critical clinical outcomes, VCE as the initial examination for OGIB appears to be optimal.

In this context, it is surprising that approximately 60 % of patients who had undergone VCE could dispense with DBE, suggesting that the strategy of initial VCE would greatly reduce the effort, burden, and cost of further examinations, including DBE. In particular, VCE for overt OGIB revealed that 25 % had bleeding sites outside the small bowel, but within the reach of upper endoscopy or colonoscopy, and that 10 % had no bleeding site throughout the intestine. The rate of presence of bleeding sites outside of the small intestine appears to be large. In fact, previous reports demonstrated that the rates ranged 12.5–28 % [12]. However, because the detection rate of small bowel lesions by VCE in our study was equivalent to that of previous reports (65 % vs. 59.4–72.5 %), [13–15] our population does not appear to be skewed by inappropriate upper GI endoscopy or colonoscopy [16]. Further investigation should be performed on this issue.

As the second relevant finding of our study, VCE could determine the appropriate insertion route for DBE. Although DBE enables endoscopic interventions including hemostasis, observation of the entire small intestine usually requires both antegrade and retrograde insertions. Because the procedure is cumbersome and requires much time and medical staffing, a predetermined appropriate insertion route for bleeding lesions would reduce the burdens on patients, endoscopists, and medical staff. Hence, VCE as the initial study, followed-by DBE, appears to be the best strategy for overt OGIB.

Li et al. suggested the ability of VCE to guide the choice of insertion route of DBE [17]. Similar to our strategy, the authors also determined the insertion route by the time point at which VCE reached the target lesion. While our point of divergence for antegrade vs. retrograde insertion was 0.5, their criterion was 0.6, (i.e., if VCE reached the target at a time 60 % or less than the total small bowel transit time, DBE was inserted by the antegrade route). The result for reaching the target lesion with the initial DBE procedure was 100 % in their study, as well as in ours, although the indication for DBE in their study was not confined to overt OGIB. If the divergence criterion of 0.6 had been adopted in our study, 2 patients who underwent retrograde DBE would have qualified for the antegrade DBE route (the indices were 0.51 and 0.58, respectively). Naturally, there may be subjects with lesions in the mid-portion of the small bowel, which DBE by either route could reach. The optimal method for choice of route for these patients should be investigated further.

Our study has limitations. First, the study design is retrospective, and the results of the single-arm strategy (initial VCE) alone were shown. Our strategy for overt OGIB, i.e., initial VCE followed by DBE, seems to be fully validated by our clinical experience, despite the drawback of the small number of the analyzed patients. For more precise evaluation of our strategy, however, studies with larger number of patients from multiple institutes and/or a prospective controlled study comparing DBE with and without prior VCE should be performed. However, such a study appears to be too risky because the effectiveness of prior VCE was obvious, and overt OGIB is sometimes fatal. Second, not all patients underwent prior upper or lower endoscopy in our hospital. Therefore, there may have been misdiagnoses of overt OGIB in our patients.

## Conclusion

In conclusion, a considerable proportion of overt OGIB patients do not require intervention including hemostasis, and can therefore avoid further invasive and costly procedures, such as DBE. The strategy of initial VCE for overt OGIB patients can efficiently identify patients who require DBE as the subsequent examination. Hence, the strategy is recommended, not only because of the safety of VCE, but also for better clinical outcomes for these patients.

## Abbreviations

DBE: Double-balloon enteroscopy
VCE: Video capsule endoscopy
OGIB: Obscure gastrointestinal bleeding

## References

[1] Raju GS, Gerson L, Das A, Lewis B (2007). American Gastroenterological Association (AGA) Institute technical review on obscure gastrointestinal bleeding. Gastroenterology.

[2] Desa LA, Ohri SK, Hutton KA, Lee H, Spencer J (1991). Role of intraoperative enteroscopy in obscure gastrointestinal bleeding of small bowel origin. Br J Surg.

[3] Costamagna G, Shah SK, Riccioni ME, Foschia F, Mutignani M, Perri V (2002). A prospective trial comparing small bowel radiographs and video capsule endoscopy for suspected small bowel disease. Gastroenterology.

[4] Iddan G, Meron G, Glukhovsky A, Swain P (2000). Wireless capsule endoscopy. Nature.

[5] Yamamoto H, Sekine Y, Sato Y, Higashizawa T, Miyata T, Iino S (2001). Total enteroscopy with a nonsurgical steerable double-balloon method. Gastrointest Endosc.

[6] Iddan GJ, Swain CP (2004). History and development of capsule endoscopy. Gastrointest Endosc Clin N Am.

[7] Yamamoto H, Kita H, Sunada K, Hayashi Y, Sato H, Yano T (2004). Clinical outcomes of double-balloon endoscopy for the diagnosis and treatment of small-intestinal diseases. Clin Gastroenterol Hepatol.

[8] Heine GD, Hadithi M, Groenen MJ, Kuipers EJ, Jacobs MA, Mulder CJ (2006). Double-balloon enteroscopy: indications, diagnostic yield, and complications in a series of 275 patients with suspected small-bowel disease. Endoscopy.

[9] Pasha SF, Leighton JA, Das A, Harrison ME, Decker GA, Fleischer DE (2008). Double-balloon enteroscopy and capsule endoscopy have comparable diagnostic yield in small-bowel disease: a meta-analysis. Clin Gastroenterol Hepatol.

[10] Akyuz U, Pata C, Kalayci M, Ozdil K, Altun H, Karip B (2012). Route selection for double balloon enteroscopy in patients with obscure gastrointestinal bleeding: experience from a single center. Turk J Gastroenterol.

[11] Pennazio M, Spada C, Eliakim R, Keuchel M, May A, Mulder CJ (2015). Small-bowel capsule endoscopy and device-assisted enteroscopy for diagnosis and treatment of small-bowel disorders: European Society of Gastrointestinal Endoscopy (ESGE) Clinical Guideline. Endoscopy.

[12] Riccioni ME, Urgesi R, Cianci R, Marmo C, Galasso D, Costamagna G (2014). Obscure recurrent gastrointestinal bleeding: a revealed mystery?. Scand J Gastroenterol.

[13] Lecleire S, Iwanicki-Caron I, Di-Fiore A, Elie C, Alhameedi R, Ramirez S (2012). Yield and impact of emergency capsule enteroscopy in severe obscure-overt gastrointestinal bleeding. Endoscopy.

[14] Nakamura M, Niwa Y, Ohmiya N, Miyahara R, Ohashi A, Itoh A (2006). Preliminary comparison of capsule endoscopy and double-balloon enteroscopy in patients with suspected small-bowel bleeding. Endoscopy.

[15] Segarajasingam DS, Hanley SC, Barkun AN, Waschke KA, Burtin P, Parent J (2015). Randomized controlled trial comparing outcomes of video capsule endoscopy with push enteroscopy in obscure gastrointestinal bleeding. Can J Gastroenterol Hepatol.

[16] Gay G, Delvaux M, Fassler I (2006). Outcome of capsule endoscopy in determining indication and route for push-and-pull enteroscopy. Endoscopy.

[17] Li X, Chen H, Dai J, Gao Y, Ge Z (2009). Predictive role of capsule endoscopy on the insertion route of double-balloon enteroscopy. Endoscopy.

